# Very low prevalence of ultrasound-detected tenosynovial abnormalities in healthy subjects throughout the age range: OMERACT ultrasound minimal disease study

**DOI:** 10.1136/annrheumdis-2021-219931

**Published:** 2021-08-18

**Authors:** Jeanette Trickey, Ilfita Sahbudin, Mads Ammitzbøll-Danielsen, Irene Azzolin, Carina Borst, Alessandra Bortoluzzi, George AW Bruyn, Philippe Carron, Coziana Ciurtin, Georgios Filippou, Jacek Fliciński, Daniela Fodor, Hélène Gouze, Marwin Gutierrez, Hilde Berner Hammer, Ellen-Margrethe Hauge, Annamaria Iagnocco, Kei Ikeda, Rositsa Karalilova, Helen Isobel Keen, Marion Kortekaas, Giuliana La Paglia, Gustavo Leon, Peter Mandl, Mihaela Maruseac, Marcin Milchert, Mohamed Atia Mortada, Esperanza Naredo, Sarah Ohrndorf, Carlos Pineda, Mads Nyhuus Bendix Rasch, Cristina Reátegui-Sokolova, Garifallia Sakellariou, Teodora Serban, Cesar A Sifuentes-Cantú, Maria S Stoenoiu, Takeshi Suzuki, Lene Terslev, Ilaria Tinazzi, Florentin Ananu Vreju, Ruth Wittoek, Maria-Antonietta D'Agostino, Andrew Filer

**Affiliations:** 1 Institute of Inflammation and Ageing, University of Birmingham, Birmingham, UK; 2 NIHR Birmingham Biomedical Reserarch Centre, University Hospitals Birmingham NHS Foundation Trust, Birmingham, UK; 3 Center for Rheumatology and Spine Diseases, Rigshospitalet, Copenhagen, Denmark; 4 Academic Rheumatology Center, MFRU, Department of Clinical and Biological Science, University of Turin, Turin, Italy; 5 Department of Dermatology, Medical University of Vienna, Vienna, Austria; 6 Section of Rheumatology, Department of Medical Sciences, University of Ferrara and Azienda Ospedaliera-Universitaria di Ferrara, Cona, Italy; 7 MC Hospital Group, Lelystad, Netherlands; 8 Reumakliniek Flevoland, Lelystad, Netherlands; 9 Department of Internal Medicine and Paediatrics, University Hospital Ghent, Ghent, Belgium; 10 VIB Center for Inflammation Research, Ghent University, Ghent, Belgium; 11 Centre for Adolescent Rheumatology, Division of Medicine, University College London, London, UK; 12 University Hospital, Rheumatology Unit, ASST Fatebenefratelli Sacco, Milan, Italy; 13 Department of Internal Medicine, Rheumatology, Diabetes, Geriatrics and Clinical Immunology, Pomeranian Medical University, Szczecin, Poland; 14 2nd Internal Medicine, UMF Iuliu Haţieganu Cluj-Napoca, Cluj-Napoca, Romania; 15 Hopital Ambroise-Pare, Boulogne-Billancourt, France; 16 Clinica Reumatologica, Università Politecnica delle Marche, Ancona, Italy; 17 Instituto Nacional de Rehabilitacion Luis Guillermo Ibarra Ibarra, Mexico, Mexico; 18 Rheumatology, Diakonhjemmet Sykehus, Oslo, Norway; 19 Faculty of Medicine, University of Oslo, Oslo, Norway; 20 Department of Rheumatology, Aarhus University Hospital, Aarhus, Denmark; 21 Department of Clinical Medicine, Aarhus University, Aarhus, Denmark; 22 Academic Rheumatology Centre, Università degli Studi di Torino, Turin, Italy; 23 Department of Allergy and Clinical Immunology, Chiba University Hospital, Chiba, Japan; 24 University Hospital Kaspela, Medical University of Plovdiv Hospital, Plovdiv, Bulgaria; 25 Medicine and Pharmacology, UWA, Perth, WA, Australia; 26 Leiden University Medical Center, Leiden, Netherlands; 27 Flevoziekenhuis, Almere, Netherlands; 28 Rheumatology Unit, Asst-Fbf-Sacco, Luigi Sacco Hospital, Milan, Italy; 29 Instituto Nacional de Rehabilitacion, Mexico, Mexico; 30 Hospital Nacional Edgardo Rebagliati Martins, Lima, Peru; 31 Department of Rheumatology, Medical University of Vienna, Vienna, Austria; 32 Department of Rheumatology, Cliniques universitaires Saint-Luc, Brussels, Belgium; 33 Department of Rheumatology Rehabilitation and Physical Medicine, Zagazig University, Zagazig, Egypt; 34 Rheumatology, Hospital Universitario Fundación Jiménez Díaz, Madrid, Spain; 35 Department of Rheumatology and Clinical Immunology, Charité–Universitätsmedizin Berlin, Campus Mitte, Humboldt–Universität zu Berlin, Freie Universität Berlin, Berlin, Germany; 36 Rheumatology, Instituto Nacional de Rehabilitación Luis Guillermo Ibarra Ibarra, Mexico, Mexico; 37 Universidad San Ignacio de Loyola, Lima, Peru; 38 Istituti Clinici Scientifici Maugeri SpA SB IRCCS, Pavia, Italy; 39 S.C. Reumatologia, ASL3 Genovese, Ospedale La Colletta, Genoa, Italy; 40 Hospital Universitario Fundación Jiménez Díaz, Madrid, Spain; 41 Department of Immunology and Rheumatology, Instituto Nacional de Ciencias Medicas y Nutricion Salvador Zubiran, Tlalpan, Mexico; 42 Institut de Recherche Expérimentale et Clinique, Université catholique de Louvain, Rheumatology Department, Cliniques universitaires Saint-Luc, Brussels, Belgium; 43 Division of Allergy and Rheumatology, Japanese Red Cross Medical Center, Shibuya, Japan; 44 Unit of Rheumatology, IRCCS Ospedale Sacro Cuore Don Calabria, Negrar, Italy; 45 Department of Rheumatology, University of Medicine and Pharmacy of Craiova, Craiova, Romania; 46 Department of Internal Medicine and Paediatrics, Ghent University, Ghent, Belgium; 47 Università Cattolica del Sacro Cuore, Fondazione Policlinico Universitario Agostino Gemelli IRCCS, Roma, Italy

**Keywords:** rheumatoid arthritis, ultrasonography, tendinopathy

## Abstract

**Objectives:**

This study aimed to determine the prevalence of ultrasound-detected tendon abnormalities in healthy subjects (HS) across the age range.

**Methods:**

Adult HS (age 18–80 years) were recruited in 23 international Outcome Measures in Rheumatology ultrasound centres and were clinically assessed to exclude inflammatory diseases or overt osteoarthritis before undergoing a bilateral ultrasound examination of digit flexors (DFs) 1–5 and extensor carpi ulnaris (ECU) tendons to detect the presence of tenosynovial hypertrophy (TSH), tenosynovial power Doppler (TPD) and tenosynovial effusion (TEF), usually considered ultrasound signs of inflammatory diseases. A comparison cohort of patients with rheumatoid arthritis (RA) was taken from the Birmingham Early Arthritis early arthritis inception cohort.

**Results:**

939 HS and 144 patients with RA were included. The majority of HS (85%) had grade 0 for TSH, TPD and TEF in all DF and ECU tendons examined. There was a statistically significant difference in the proportion of TSH and TPD involvement between HS and subjects with RA (HS vs RA p<0.001). In HS, there was no difference in the presence of ultrasound abnormalities between age groups.

**Conclusions:**

Ultrasound-detected TSH and TPD abnormalities are rare in HS and can be regarded as markers of active inflammatory disease, especially in newly presenting RA.

**Key messages**

**What is already known about this subject?**

- Little is known about the prevalence of sonographic tenosynovial abnormalities in healthy subjects (HS) across the age range.

**What does this study add?**

- This is the largest cohort of healthy subjects with tendons scanned by ultrasound.
- There is very low prevalence of tendon synovial hypertrophy or power Doppler abnormalities in tendons of HS even in old age.
- Ultrasound-detected inflammation in digit flexor and extensor carpi ulnaris tendons in patients suspected to be in the early stages of rheumatoid arthritis (RA) should not be discounted as physiological, even in older age.

**How might this impact on clinical practice or future developments?**

- Ultrasound-detected tenosynovial abnormalities can be regarded as robust findings in the clinical management of early RA.

## Introduction

Tenosynovitis (TS) of hand and wrist tendons is common in early untreated inflammatory polyarthritis.^1^ However, clinical examination alone may not detect this pathology,^2^ especially as conventional rheumatoid arthritis (RA) disease activity scoring systems focus on joints, not tendons. The use of MRI and ultrasound examination is more sensitive and has shown that the prevalence of detecting TS in patients with early RA is higher than by physical examination alone.^3^

There has been extensive focus on the sensitivity and role of ultrasound in detecting subclinical synovial inflammation.^4 5^ Ultrasound has been shown to be highly sensitive in the detection of tenosynovial inflammation, with recent studies demonstrating that ultrasound-detected hand and wrist TS has a role in predicting outcome in early RA and flare in clinical remission.^6 7^

Although recent studies using MRI have focused on the prevalence of tendon abnormalities in healthy subjects (HS),^8^ there are limited data on the prevalence of ultrasound-detected ‘TS’ abnormalities in HS, with data arising from small comparison cohorts (ie, case–control studies focused on patients with rheumatic diseases). Furthermore, current studies were not focused on the prevalence of sonographic tendon abnormalities in HS within the age range of 40–70 years when RA commonly presents.^9^ The prevalence of such abnormalities therefore remains unknown in this group.

The objective of this Outcome Measures in Rheumatology (OMERACT) ultrasound study was therefore to determine the prevalence of ultrasound-detected tendon abnormalities characterising the presence of TS in HS according to the age range.

## Methods

Adult HS (18–80 years) were recruited between August 2017 and December 2018 in 23 ultrasound centres in 14 countries with experience of participating in OMERACT ultrasound studies. To ensure a wide range of age coverage, recruitment was obtained from a large range of populations: university or hospital research staff, health service workers, students, volunteers from local advertising or national cohorts such as the Birmingham 1000 Elders group^10^ in the UK. Exclusion criteria were current or previous history of any form of inflammatory arthritis, joint trauma of hands or wrist in the previous month; hand or wrist pain of o≥10/100 on the Visual Analogue Scale; hand osteoarthritis according to American College of Rheumatology (ACR) criteria^11^; history of infection; and recent or current use of medications that may affect ultrasound assessment (see online supplemental table 1). An additional 12 HS were excluded after data collection but before ultrasound analysis due to autoimmune, infectious or musculoskeletal conditions identified from medical history that could confound the results. Demographic data including body mass index (BMI) were collected. Metacarpophalangeal, proximal interphalangeal, metatarsophalangeal and wrist joints were clinically examined by an independent assessor in each centre, and subjects were excluded if synovitis was found.

10.1136/annrheumdis-2021-219931.supp2 Supplementary data

Ultrasound assessment of bilateral digit flexors (DFs) 1–5 and extensor carpi ulnaris (ECU) tendons was performed using a multiplanar approach. The presence of hypoechoic tenosynovial hypertrophy (TSH) and power Doppler signal within tenosynovial power Doppler (TPD) was defined and graded using the OMERACT ultrasound scoring system for TS in RA.^12^ The ungraded presence of tenosynovial effusion (TEF) was recorded. Adequate gel was used to avoid compression. Views were recorded according to European League Against Rheumatism (EULAR) standard reference scan guidelines.^13^ Musculoskeletal specific preset parameters were used to optimise imaging for greyscale and power Doppler and reduce variability. Details of probes, machines and experience of sonographers in all centres can be found in online supplemental table 2. Quality and grading of recorded images were confirmed by a review of all images for the first HS recruited in each centre by an experienced blinded independent assessor (IS) in the hub centre. Any disagreement was then fed back to the centre and consensus was achieved to ensure reliability in subsequent scans.

Data for a comparison cohort of DMARD-naive patients presenting as patients with new early arthritis with RA fulfilling ACR-EULAR 2010^14^ and/or 1987 criteria^15^ at presentation were extracted from the Birmingham Early Arthritis (BEACON) inception cohort.^6^ The following data were collected: 68 tender and 66 swollen clinical counts, age, sex, symptom duration, early morning stiffness duration, medication, erythrocyte sedimentation rate (ESR), C reactive protein (CRP), rheumatoid factor and anti-citrullinated protein antibody status. This cohort underwent identical baseline tendon ultrasound assessment except for the presence of TEF.

### Statistical analysis

Statistical analysis was performed using IBM SPSS Statistics V.26. Significance for the binary variable gender was assessed using Fisher’s exact test. The continuous variables age and BMI (for all subjects) and early morning stiffness, CRP and joint counts (for patients with RA) were not normally distributed; significance was therefore assessed using the Kruskal-Wallis test. The tendon gradings were dichotomised into either present (grades 1–3) or absent (grade 0). Fisher’s exact test was used to compare the proportions of grade 1–3 TSH, TPD or TEF between age groups in HS, and between HS and patients with RA.

## Results

One thousand and forty-nine HS were recruited and 939 HS were included after exclusions of subjects with protocol deviations (see flowchart in online supplemental figure 1). Baseline data for 144 patients with RA were randomly extracted from the BEACON database and matched with a cohort of 144 HS by age, sex and smoking status where possible. Table 1 shows the demographic and ultrasound characteristics of the two populations. Full ultrasound grading results are available in online supplemental table 2 and example of grading in online supplemental figure 2.

10.1136/annrheumdis-2021-219931.supp1 Supplementary data

**Table 1:** Demographics and tendon changes (grade 1–3 TSH and power Doppler) for HS and patients with RA

	HS Y 18–39 year	HS M 40–59 year	HS O ≥60 years	HS Y/M/O P value	RA	RA versus age-matched and sex-matched HS* P value
n	405	350	184		144	
Age (years), median (IQR)	29 (25–33)	49 (44–54)	68 (62–72)	<0.001	54 (45–67)	1.000
Female, n (%)	268 (66.2)	285 (81.4)	117 (63.6)	<0.001	106 (73.6)	0.924
BMI, median (IQR)	23 (22–24)	25 (21–28)	26 (23–28)	<0.001	27 (24–32)	<0.001
Smoking						
Never (%)	316 (78)	241 (68)	115 (63)		68 (47)	0.021
Ever (%)	88 (22)	109 (31)	66 (36)		75 (52)	
Current (%)	47 (12)	56 (16)	12 (7)		28 (19)	
EMS (min), median (IQR)	n/a	n/a	n/a	n/a	60 (15–120)	n/a
Symptom duration (weeks), median (IQR)	n/a	n/a	n/a	n/a	26 (13–52)	n/a
CRP (mg/L), median (IQR)	n/a	n/a	n/a	n/a	7 (3–20)	n/a
DAS28 CRP, median (IQR)	n/a	n/a	n/a	n/a	5.1 (4.1–5.8)	n/a
Tender joint,† median (IQR)	0 (0–0)	0 (0–0)	0 (0–0)	n/a	17 (11–27)	<0.001
Swollen joint,† median (IQR)	0 (0–0)	0 (0–0)	0 (0–0)	n/a	6 (3–11)	<0.001
DF 1 TSH grade ≥1, n (%)	1 (0.1)	0 (0)	1 (0.3)	0.490	15 (5.2)	<0.001
DF 2 TSH grade ≥1, n (%)	1 (0.1)	2 (0.3)	0 (0)	0602	50 (17.3)	<0.001
DF 3 TSH grade ≥1, n (%)	2 (0.2)	1 (0.1)	2 (0.6)	0.432	50 (17.3)	<0.001
DF 4 TSH grade ≥1, n (%)	2 (0.2)	1 (0.1)	1 (0.3)	1.000	28 (9.8)	<0.001
DF 5 TSH grade ≥1, n (%)	1 (0.1)	4 (0.6)	0 (0)	0.220	36 (12.5)	<0.001
ECU TSH grade ≥1, n (%)	7 (0.9)	9 (1.3)	1 (0.3)	0.293	65 (22.6)	<0.001
DF 1 TPD grade ≥1, n (%)	1 (0.1)	0 (0)	1 (0.3)	0.490	10 (3.5)	0.002
DF 2 TPD grade ≥1, n (%)	0 (0)	1 (0.1)	0 (0)	0.568	36 (12.6)	<0.001
DF 3 TPD grade ≥1, n (%)	1 (0.1)	0 (0)	0 (0)	1.000	40 (13.9)	<0.001
DF 4 TPD grade ≥1, n (%)	0 (0)	0 (0)	1 (0.3)	0.194	20 (7)	<0.001
DF 5 TPD grade ≥1, n (%)	0 (0)	0 (0)	0 (0)	n/a	23 (8.1)	<0.001
ECU TPD grade≥1, n (%)	0 (0)	0 (0)	0 (0)	n/a	62 (21.7)	<0.001
Total grade tendon score,‡ mean (range)	0.04 (0-2)	0.05 (0-4)	0.04 (0-2)		3.02 (0-21)	
Total count of tendons grade ≥1,§ mean (range)	0.03 (0-2)	0.05 (0-4)	0.03 (0-2)		1.69 (0-11)	
Individuals with grade ≥1 TSH, n (%)	12 (3.0)	10 (2.8)	4 (2.1)		76 (52.8)	
Individuals with grade ≥1 TPD, n (%)	2 (0.5)	1 (0.3)	2 (1.1)		63 (43.7)	
Individuals with grade ≥1 TEF, n (%)	50 (12.2)	46 (13.2)	29 (15.8)		n/a	

*RA and HS age matched and sex matched to compare ultrasound graded tendon findings.

†Patients with RA had 66/68 joint counts; HS had joint counts of MCPs, PIPs, wrists and MTPs.

‡Total grade tendon score is the per patient sum of all grades of TSH and TPD tendon abnormalities.

§ Total count of tendons grade ≥1 includes TSH and TPD.

BMI, body mass index; CRP, C reactive protein; DAS28, Disease Activity Score in 28 joints; DF, digit flexor; ECU, extensor carpi ulnaris; EMS, early morning stiffness; HS, healthy subjects; M, middle; MCP, metacarpophalangeal; MTP, metatarsophalangeal; O, old; PIP, proximal interphalangeal; RA, rheumatoid arthritis; TEF, tenosynovial effusion; TPD, tendon power Doppler; TSH, tenosynovial hypertrophy; Y, young.

### Healthy subjects

The median age of HS was 43 years (30–57). HS were grouped into three age groups: HS Y (young, 18–39 years) HS M (middle, 40–59 years) and HS O (old, 60–80 years) for analysis. The majority of volunteer HS were healthcare professionals (423, 45.0%). Other occupational groups included clerical staff (156, 16.6%), students (95, 10.1%), manual workers (68, 7.2%) and teachers (34, 3.6%).

A total of 11 237 tendons were scanned; 98% of these tendons were grade 0 for TSH, TPD and TEF (online supplemental table 3). The distribution of tendon abnormalities, when found, was symmetrical with no significant difference between right and left hands (online supplemental table 4). TEF was more frequently detected than TSH or TPD (p<0.001) (online supplemental table 5).

**Figure 1:**
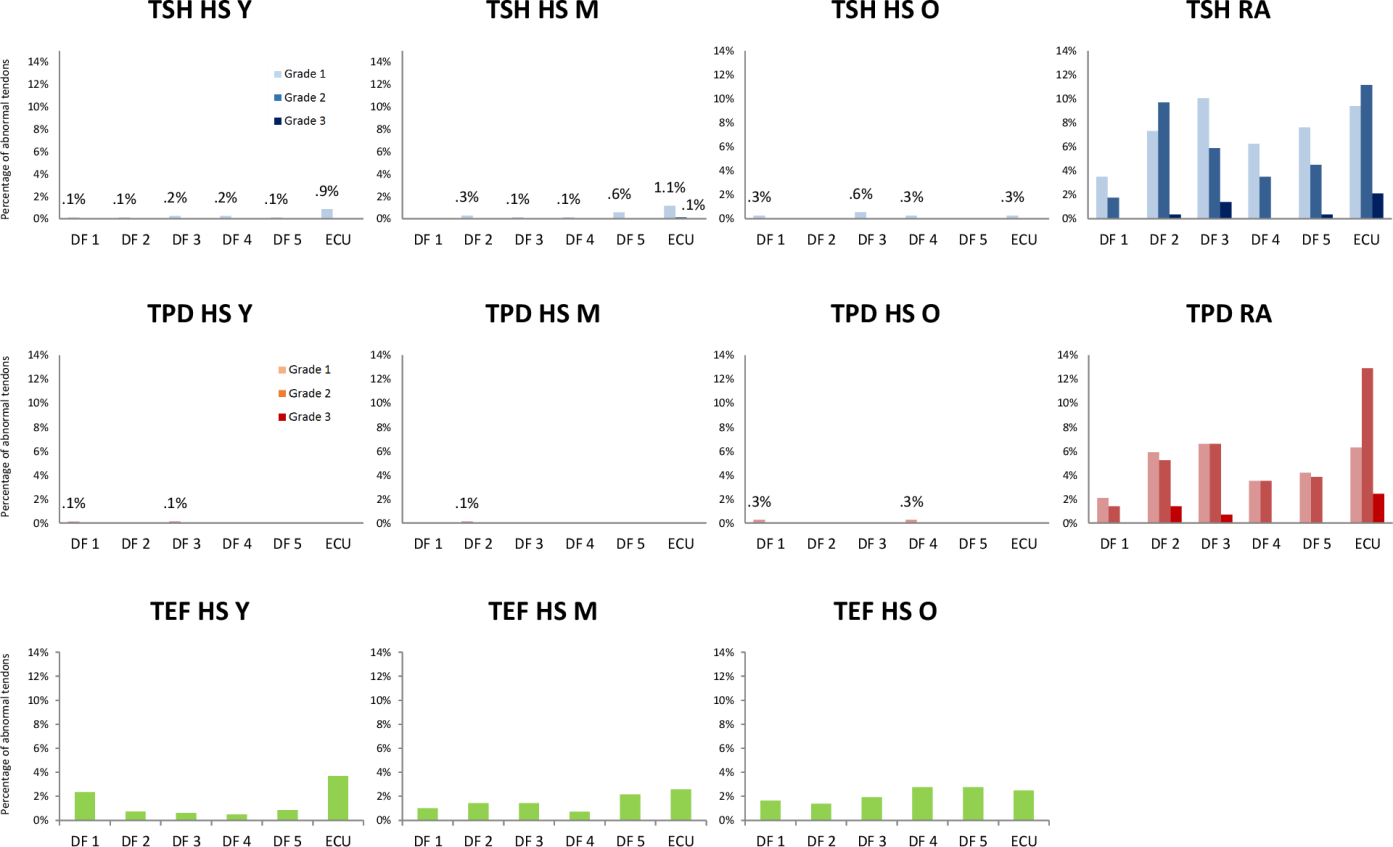
Percentage of tendons with grade 1–3 TSH and TPD, and presence of TEF in DF tendons 1–5 and ECU for HS according to age groups, compared with patients with RA. TEF measured only in HS. HS Y, 18–39 years; HS M, 40–59 years; HS O, 60–80 years. DF, digit flexor; ECU, extensor carpi ulnaris tendon; HS, healthy subjects; M, middle; O, old; RA, rheumatoid arthritis; TEF, tenosynovial effusion; TPD, tenosynovial power Doppler; TSH, tenosynovial hypertrophy; Y, young.

The majority (791/939, 84.2%) of HS presented grade 0 overall for all ultrasound lesions examined (TSH, TPD and TEF) in all DF 1–5 and ECU tendons. In particular 99% (931/939) of HS had grade 0 for TPD in all tendons scanned. There were no statistically significant differences between age groups (table 1 and figure 1).

Abnormalities were detected in 148 individuals across 939 HS and were of grade 1 severity, with the exception of one grade 2 for TSH in an ECU tendon. The ECU tendons had significantly more grade ≥1 for TSH than the DF 1–5 tendons (p<0.05) (online supplemental table 6).

There was no statistically significant difference in the proportion of TSH or TPD ≥1 in HS with manual professions, or in those who practice sports or hobbies which may have high impact on the upper limbs (online supplemental tables 7 and 8).

### Patients with RA

Patients with RA were matched with 144 HS by age (within 2 years) and sex, and with smoking status in 116/144 HS. TS as defined by TSH and power Doppler grade ≥1 in DF and ECU tendons was more prevalent in patients with RA (52.8%) compared with HS (0.9%). There were significantly more TSH and TPD grade ≥1 detected in patients with RA compared with age-matched and sex-matched HS (p=0.002 to <0.001) (online supplemental table 9).

## Discussion

Our study is the first to assess tendon involvement in large numbers of HS, encompassing the age incidence of RA with 367 HS over 50 years, and showing a very low prevalence of abnormal findings. The few abnormalities observed were almost exclusively grade 1 in severity. Due to the large population assessed, we provide conclusive data validating and expanding on the findings of existing studies with few HS.^16–18^

TEF was more prevalent than TSH or TPD in HS. Although MRI studies have suggested TEF to be almost ubiquitous in DF tendons in HS,^19^ we have shown that ultrasound detects smaller numbers: less than 2% of DF tendons even in the older age group. Visualisation of tendons in two dimensions is the most likely cause of this difference. Tenosynovial abnormalities on ultrasound were significantly more prevalent in early RA compared with matched HS.

By explicitly selecting only subjects with minimal joint pain and without overt osteoarthritis, and by using a non-random recruitment strategy to ensure inclusion of an older cohort, HS in this study may have fewer tendon changes than an unselected general population of 60–80 year olds. However, it was not our purpose to document the presence of tendon abnormalities in unselected primary or secondary care early arthritis clinics or in osteoarthritis, but to assess if HS with no symptoms may have ultrasound inflammatory abnormalities. The lack of a formal reliability study which would have been logistically difficult in such a large study, and the consecutive, not blinded recruitment may be seen as potential limitations. We mitigated these by designing a blinded central regrading strategy of the first HS scan performed by each centre.^20^

The very low prevalence of TSH and TPD across a large age range in HS suggests that these findings can be seen as potentially pathological, and not simply the consequence of ageing, by health professionals performing ultrasound in early arthritis or disease management clinics. The interpretation of such findings should depend on the clinical context. In addition, DF and ECU tendons can be easily examined during routine ultrasound examination and so could be included in abbreviated scanning protocols.

10.1136/annrheumdis-2021-219931.supp3 Supplementary data

## Data Availability

All data relevant to the study are included in the article or uploaded as supplemental information. Anonymised data are available on request from the authors.

## References

[1] Hmamouchi I , Bahiri R , Srifi N , et al . A comparison of ultrasound and clinical examination in the detection of flexor tenosynovitis in early arthritis. BMC Musculoskelet Disord 2011;12:91. 10.1186/1471-2474-12-91 21549008 PMC3112434

[2] Hamdi W , Miladi S , Cherif I , et al . AB0311 Superiority of Ultrasound over Clinical Examination in Detecting Tenosynovitis in Rheumatoid Arthritis. Ann Rheum Dis 2015;74:997.3–8. 10.1136/annrheumdis-2015-eular.5265

[3] Wakefield RJ , O'Connor PJ , Conaghan PG , et al . Finger tendon disease in untreated early rheumatoid arthritis: a comparison of ultrasound and magnetic resonance imaging. Arthritis Rheum 2007;57:1158–64. 10.1002/art.23016 17907233

[4] Naredo E et al . Assessment of inflammatory activity in rheumatoid arthritis: a comparative study of clinical evaluation with grey scale and power Doppler ultrasonography. Ann Rheum Dis 2005;64:375–81. 10.1136/ard.2004.023929 15708891 PMC1755396

[5] Dale J , Stirling A , Zhang R , et al . Targeting ultrasound remission in early rheumatoid arthritis: the results of the TaSER study, a randomised clinical trial. Ann Rheum Dis 2016;75:1043–50. 10.1136/annrheumdis-2015-208941 27026689

[6] Sahbudin I , Pickup L , Nightingale P , et al . The role of ultrasound-defined Tenosynovitis and synovitis in the prediction of rheumatoid arthritis development. Rheumatology 2018;57:1243–52. 10.1093/rheumatology/key025 29618136 PMC6037116

[7] Filippou G , Sakellariou G , Scirè CA , et al . The predictive role of ultrasound-detected Tenosynovitis and joint synovitis for flare in patients with rheumatoid arthritis in stable remission. Results of an Italian multicentre study of the Italian Society for rheumatology group for ultrasound: the starter study. Ann Rheum Dis 2018;77:1283–9. 10.1136/annrheumdis-2018-213217 29886430

[8] Mangnus L , van Steenbergen HW , Reijnierse M , et al . Magnetic resonance Imaging-Detected features of inflammation and erosions in symptom-free persons from the general population. Arthritis Rheumatol 2016;68:2593–602. 10.1002/art.39749 27213695

[9] Muilu P , Rantalaiho V , Kautiainen H , et al . Increasing incidence and shifting profile of idiopathic inflammatory rheumatic diseases in adults during this millennium. Clin Rheumatol 2019;38:555–62. 10.1007/s10067-018-4310-0 30259249

[10] Lord J . The Birmingham 1000 Elders - playing a leading role in Healthy Ageing Research, 2020. Available: https://www.birmingham.ac.uk/research/inflammation-ageing/research/1000-elders/elders.aspx [Accessed 11 Jul 2021].

[11] Altman R , Alarcón G , Appelrouth D , et al . The American College of rheumatology criteria for the classification and reporting of osteoarthritis of the hand. Arthritis Rheum 1990;33:1601–10. 10.1002/art.1780331101 2242058

[12] Naredo E , D'Agostino MA , Wakefield RJ , et al . Reliability of a consensus-based ultrasound score for tenosynovitis in rheumatoid arthritis. Ann Rheum Dis 2013;72:1328–34. 10.1136/annrheumdis-2012-202092 22984169

[13] Backhaus M , Burmester GR , Gerber T , et al . Guidelines for musculoskeletal ultrasound in rheumatology. Ann Rheum Dis 2001;60:641–9. 10.1136/ard.60.7.641 11406516 PMC1753749

[14] Aletaha D , Neogi T , Silman AJ , et al . 2010 rheumatoid arthritis classification criteria: an American College of Rheumatology/European League against rheumatism collaborative initiative. Ann Rheum Dis 2010;69:1580–8. 10.1136/ard.2010.138461 20699241

[15] Arnett FC , Edworthy SM , Bloch DA , et al . The American rheumatism association 1987 revised criteria for the classification of rheumatoid arthritis. Arthritis Rheum 1988;31:315–24. 10.1002/art.1780310302 3358796

[16] Guerini H , Pessis E , Theumann N , et al . Sonographic appearance of trigger fingers. J Ultrasound Med 2008;27:1407–13. 10.7863/jum.2008.27.10.1407 18809950

[17] Micu MC , Fodor D , Micu R , et al . Pregnant versus non-pregnant healthy subjects - a prospective longitudinal musculoskeletal ultrasound study concerning the spectrum of normality. Med Ultrason 2018;20:319–27. 10.11152/mu-1630 30167585

[18] Piga M , Gabba A , Congia M , et al . Predictors of musculoskeletal flares and Jaccoud׳s arthropathy in patients with systemic lupus erythematosus: a 5-year prospective study. Semin Arthritis Rheum 2016;46:217–24. 10.1016/j.semarthrit.2016.04.005 27238877

[19] Agten CA , Rosskopf AB , Jonczy M , et al . Frequency of inflammatory-like MR imaging findings in asymptomatic fingers of healthy volunteers. Skeletal Radiol 2018;47:279–87. 10.1007/s00256-017-2808-1 29110050

[20] D'Agostino M-A , Wakefield RJ , Berner-Hammer H , et al . Value of ultrasonography as a marker of early response to abatacept in patients with rheumatoid arthritis and an inadequate response to methotrexate: results from the appraise study. Ann Rheum Dis 2016;75:1763–9. 10.1136/annrheumdis-2015-207709 26590174 PMC5036216

