# Supplementary material for: Very low prevalence of ultrasound-detected tenosynovial abnormalities in healthy subjects throughout the age range: OMERACT ultrasound minimal disease study
Source: Ann Rheum Dis. 2021 Aug 18;81(2):232–6. doi: 10.1136/annrheumdis-2021-219931 (PMC8762026; doi:10.1136/annrheumdis-2021-219931)
Supplement: Supplementary data [file annrheumdis-2021-219931supp002.pdf]

Supplementary table 1: inclusion and exclusion criteria for healthy subjects

**Inclusion criteria**
Age 18-80 years
**Exclusion criteria**
Previous/current inflammatory joint disease (including crystal arthropathy)
Visual analogue score (VAS) for joint pain > 10/100.
Any history of joint trauma in the last month.
Fulfilling hand osteoarthritis ACR criteria
Any clinical joint inflammation as identified by a physician.
Previous or current inflammatory bowel disease.
History of culture-proven enteric and/or genitourinary infection in the last month
Current or previous corticosteroids use in the last 4 weeks.
Current non-steroidal anti-inflammatory use.

Supplementary table 2: Ultrasound machines and transducers used by centres

**Centre**	**Contributors**	**Years of ultrasound experience**	**Ultrasound qualifications**	**Machine**	**Linear Transducer**
**Institute of Inflammation and Ageing, University of Birmingham, UK**	Andrew Filer	15	EULAR teach the teacher<br>EULAR level 2 (max)	GE Logiq E9	8-18MHz;<br>6-15MHz
	Ilfita Sahbudin	9	MSc in Musculoskeletal Ultrasound, University of Bournemouth		
	Jeanette Trickey	6	BSR Basic Ultrasound Course		
**University College London, UK**	Coziana Ciurtin	10	EULAR level 2 (max)	GE Logiq E8	8-15MHz
**Hôpital Ambroise Paré, Paris, France**	Maria-Antonietta D'agostino	25	EFSUMB level 3 (max),<br>EULAR level 2 (max)	ESAOTE MyLab70 XVG	6-18MHz<br>PD 11 MHz,<br>PRF 750Hz
	Hélène Gouze	6	French Musculoskeletal Ultrasound Course,<br>EULAR Ultrasound intermediate Course		
**Department of Rheumatology Cliniques Universitaires Saint-Luc, Brussels Belgium**	Maria Stoenoiu	15	EFSUMB level 3 (max),<br>EULAR level 2 (max)	GE Logic E9	M L6-15;<br>L8-18i
	Mihaela Maruseac	5	EULAR intermediate,<br>EFSUMB level 2		

Trickey J, *et al. Ann Rheum Dis* 2022; 781:232–236. doi: 10.1136/annrheumdis-2021-219931

**Ghent University, Belgium**	Ruth Wittoek	15	EULAR level 2	ESAOTE MyLab 60	
	Philippe Carron	13	EULAR level 3		
**University of Ferrara, Italy**	Alessandra Bortoluzzi	12	Basic EULAR ultrasound course; advanced MSUS course endorsed by the Italian Society for Rheumatology	ESAOTE MyLab 70XVG	14–18 MHz
**University of Ferrara, Italy**	Georgios Filippou	20	EULAR level 2 (max)	Samsung RS80A	4-18 MHz; 3-12MHz
**Università degli Studi di Torino, Turin, Italy**	Annamaria Iagnocco	37	EFSUMB level 3 (max), EULAR level 2 (max)	ESAOTE MyLab8	L4-15 (4-15 MHz); LA 435 (6-18MHz)
	Teodora Șerban	11	EFSUMB level 1, EULAR level 2 (max), Romanian Ministry of Health Certified Sonographer		
	Irene Azzolin	5	Musculoskeletal Ultrasound in Rheumatology -EULAR Basic Course		
**University of Pavia, Italy**	Garifallia Sakellariou	11	none	ESAOTE Mylab 70 XVG	ESAOTE LA435 (6-18 MHz) PRF 0.75;
**Sacro Cuore Hospital, Negrar, Verona, Italy**	Ilaria Tinazzi	16	EULAR intermediate ultrasound course in 2007	ESAOTE MyLabClassC	10-18 MHz PRF 750 Hz
**Copenhagen University Hospital, Denmark**	Lene Terslev	22	EFSUMB level 3 (max), EULAR level 2 (max) Danish Rheumatology Association level 5 (max)	GE Logiq E9	ML 6-15 Colour Doppler (CD) 7.5 MHz, PRF 0.4
	Mads Ammitzball Danielsen	10	EFSUMB level 2, EULAR level 1, Danish Rheumatology Association level 4		
**Aarhus University Hospital, Denmark**	Ellen-Margrethe Hauge	16	Danish Rheumatology Association level 3 (max)	Hitachi Noblus	18-5 L64 Colour Doppler (CD) 6.5 MHz
	Mads Nyhuus Bendix Rasch	10	Danish Rheumatology Association level 4		
**Diakonhjemmet**	Hilde Berner Hammer	20	EFSUMB level 3 (max),	GE Logiq E9	6-15 MHz

**Hospital, Oslo, Norway**			EULAR level 2 (max) EULAR faculty US courses		
**Leiden University Medical Center, The Netherlands.**	Marion Kortekaas	16	EULAR level 2 (max), US level of the Dutch Rheumatology Association (max)	GE logic E9	5-18 MHz
	Sarah Ohrndorf	14	EULAR level 2 (max)		
**Pomeranian Medical University, Szczecin, Poland**	Marcin Milchert	13	Large Vessel Vasculitis Ultrasound courses attendances and tutorship (Hospital of Southern Norway Trust, Kristiansand, Norway) Tutorship of Large Vessel Vasculitis Ultrasound courses Southend Hospital, UK and others. Certificates in Vascular Doppler Sonography and MS Sonography of Polish Ultrasonography Society and Polish Rheumatology Society	Phillips Epiq 5	18 MHz
	Jacek Fliciński	12	Level 1 of EULAR Competency Assessment in MSUS Teach the Teachers course Certificate of Proficiency Musculoskeletal Sonography Polish Rheumatological Society		
**Medical University of Vienna, Vienna, Austria**	Peter Mandl	16	EFSUMB level 3 (max), EULAR level 2 (max)	GE Logiq E9	6-15MHz, 8-18MHz
	Carina Borst	3	none		
**Iuliu Hatieganu University of Medicine and Pharmacy, Cluj-Napoca, Romania**	Daniela Fodor	25	EFSUMB level 3, EULAR level 2	GE S7	L8-18I
**University of Medicine and Pharmacy, Craiova,**	Florentin Vreju	15	EULAR intermediate course, EULAR advanced course, EULAR teach-the-	ESAOTE Mylab 25 Gold	18MHz

**Romania**			teachers course, EFSUMB		
**Medical University of Plovdiv, Bulgaria.**	Rositsa Karalilova	13	EULAR level 2 (maximum), EULAR Certificate for Ultrasound Trainers in Rheumatology	ESAOTE MyLab 7	3-18 MHz
**Hospital Universitario Fundación Jiménez Díaz, Madrid, Spain.**	Esperanza Naredo	25	EFSUMB level 3 (max, EULAR level 2 (max)	GE Logiq E9	ML6-15 (6-15MHz)
	Cesar Sifuentes-Cantu	5	Certifications by the Mexican College of Rheumatology		
	Giuliana M.C. La Paglia	6	EULAR intermediate ultrasound course, EFSUMB level 1		
**Instituto Nacional de Rehabilitacion, Mexico City, Mexico**	Carlos Pineda	23	PANLAR Level 3, ECOMER Level 3 (max)	GE Logiq e	8-18 MHz
	Marwin Gutierrez	17	EFSUMB Level 3; EULAR Level 2; PANLAR Level 3		
	Gustavo Leon				
	Cristina Reategui-Sokolova	6	Fellowships in Peruvian and Mexican hospitals.		
**Zagazig University, Egypt**	Mohamed Mortada	17	EULAR advanced course of musculoskeletal ultrasonography	Hitachi Aloka F37	18 MHz
**Japanese Red Cross Medical Center, Tokyo, Japan**	Takeshi Suzuki	17	EULAR intermediate course, EULAR teach-the-teachers course, JCR-certified sonographer	HI VISION Avius	5-18 MHz PD frequency 7.5MHz, PRF 800Hz
**Chiba University Hospital, Japan**	Kei Ikeda	18	EULAR intermediate course, EULAR advanced course, EULAR teach-the-teachers course, JCR-certified sonographer	HI VISION Avius; HI VISION Ascendus	EUP-L75 (5-18MHz)

Supplementary table 3: complete ultrasound grade results for tenosynovial hypertrophy, tenosynovial Power Doppler and tendon sheath effusion in Heathy Subjects and patients with Rheumatoid Arthritis

Healthy Subjects

	HS Y (18-39 years)			HS M (40-59 years)			HS O (60-80 years)			p value* HS Y vs M vs O
	G 1 n (%)	G 2 n (%)	G 3 n (%)	G 1 n (%)	G 2 n (%)	G 3 n (%)	G 1 n (%)	G 2 n (%)	G 3 n (%)	
**TSH**										
**DF 1**	1 (0.1)	0 (0.0)	0 (0.0)	0 (0.0)	0 (0.0)	0 (0.0)	1 (0.3)	0 (0.0)	0 (0.0)	0.490
**DF 2**	1 (0.1)	0 (0.0)	0 (0.0)	2 (0.3)	0 (0.0)	0 (0.0)	0 (0.0)	0 (0.0)	0 (0.0)	0602
**DF 3**	2 (0.2)	0 (0.0)	0 (0.0)	1 (0.1)	0 (0.0)	0 (0.0)	2 (0.6)	0 (0.0)	0 (0.0)	0.432
**DF 4**	2 (0.2)	0 (0.0)	0 (0.0)	1 (0.1)	0 (0.0)	0 (0.0)	1 (0.3)	0 (0.0)	0 (0.0)	1.000
**DF 5**	1 (0.1)	0 (0.0)	0 (0.0)	4 (0.6)	0 (0.0)	0 (0.0)	0 (0.0)	0 (0.0)	0 (0.0)	0.220
**ECU**	7 (0.9)	0 (0.0)	0 (0.0)	8 (1.1)	1 (0.1)	0 (0.0)	1 (0.3)	0 (0.0)	0 (0.0)	0.293
**TPD**										
**DF 1**	1 (0.1)	0 (0.0)	0 (0.0)	0 (0.0)	0 (0.0)	0 (0.0)	1 (0.3)	0 (0.0)	0 (0.0)	0.490
**DF 2**	0 (0.0)	0 (0.0)	0 (0.0)	1 (0.1)	0 (0.0)	0 (0.0)	0 (0.0)	0 (0.0)	0 (0.0)	0.568
**DF 3**	1 (0.1)	0 (0.0)	0 (0.0)	0 (0.0)	0 (0.0)	0 (0.0)	0 (0.0)	0 (0.0)	0 (0.0)	1.000
**DF 4**	0 (0.0)	0 (0.0)	0 (0.0)	0 (0.0)	0 (0.0)	0 (0.0)	1 (0.3)	0 (0.0)	0 (0.0)	0.194
**DF 5**	0 (0.0)	0 (0.0)	0 (0.0)	0 (0.0)	0 (0.0)	0 (0.0)	0 (0.0)	0 (0.0)	0 (0.0)	n/a
**ECU**	0 (0.0)	0 (0.0)	0 (0.0)	0 (0.0)	0 (0.0)	0 (0.0)	0 (0.0)	0 (0.0)	0 (0.0)	n/a

	Present n (%)	Present n (%)	Present n (%)	
**TEF**				
**DF 1**	19 (2.3)	7 (1.0)	6 (1.7)	<0.001
**DF 2**	6 (0.7)	10 (1.4)	5 (1.4)	0.001
**DF 3**	5 (0.6)	10 (1.4)	7 (1.9)	<0.001
**DF 4**	4 (0.5)	5 (0.7)	10 (2.8)	<0.001
**DF 5**	7 (0.8)	15 (2.1)	10 (2.8)	<0.001
**ECU**	30 (3.7)	18 (2.6)	9 (2.5)	0.001

HS, healthy subject; G, grade; THS, tenosynovial hypertrophy; TPD, power Doppler within the tendon sheath; TEF, tenosynovial effusion; DF, digit flexor tendon; ECU, extensor carpi ulnaris tendon. * Fisher's exact test

Patients with Rheumatoid Arthritis

	Grade 1 n (%)	Grade 2 n (%)	Grade 3 n (%)		Grade 1 n (%)	Grade 2 n (%)	Grade 3 n (%)
**TSH**				**TPD**			
**DF 1**	10 (3.5)	5 (1.8)	0 (0.0)	DF 1	6 (2.1)	4 (1.4)	0 (0.0)
**DF 2**	21 (7.3)	28 (9.7)	1 (0.4)	DF 2	17 (5.9)	15 (5.3)	4 (1.4)
**DF 3**	29 (10.1)	17 (5.9)	4 (1.4)	DF 3	19 (6.6)	19 (6.6)	2 (0.7)
**DF 4**	18 (6.3)	10 (3.5)	(0.0)	DF 4	10 (3.5)	10 (3.5)	0 (0.0)
**DF 5**	22 (7.6)	13 (4.5)	1 (0.4)	DF 5	12 (4.2)	11 (3.9)	0 (0.0)
**ECU**	27 (9.4)	32 (11.2)	6 (2.1)	ECU	18 (6.3)	37 (12.9)	7 (2.5)

TSH, tenosynovial hypertrophy; TPD, power Doppler within tendon sheath; TEF, tenosynovial effusion

Trickey J, *et al. Ann Rheum Dis* 2022; 781:232–236. doi: 10.1136/annrheumdis-2021-219931

Supplementary Table 4: Healthy subjects and RA patients with grade 0 for ultrasound findings

	TSH all grade 0 n (%)	TPD all grade 0 n (%)	TEF all grade 0 n (%)	TSH, TPD and TEF all grade 0 n (%)
**Healthy subjects n= 939**	907 (96.6)	931 (99.1)	808 (86.0)	791 (84.3)
**RA patients n= 144**	68 (47.2)	81 (56.3)	n/a	n/a

HS, healthy subjects; RA, patients with Rheumatoid Arthritis; TSH, tenosynovial hypertrophy; TPD, power Doppler within tendon sheath; TEF, tenosynovial effusion

Supplementary table 5: Distribution ultrasound findings of grade ≥ 1 in left and right tendons in healthy subjects

	HS Y (18-39 yrs)		HS M (40-59 yrs)		HS O (60-80 yrs)		All age groups		
	Left n (%)	Right n (%)	Left n (%)	Right n (%)	Left n (%)	Right n (%)	Left n (%)	Right n (%)	p value* L vs R
**TSH**									
**DF1**	0 (0.0)	1 (0.2)	0 (0.0)	0 (0.0)	1 (0.5)	0 (0.0)	1 (0.1)	1 (0.1)	1.000
**DF2**	1 (0.2)	0 (0.0)	0 (0.0)	2 (0.6)	0 (0.0)	0 (0.0)	1 (0.1)	2 (0.2)	1.000
**DF3**	1 (0.2)	1 (0.2)	1 (0.3)	0 (0.0)	1 (0.5)	1 (0.6)	3 (0.3)	2 (0.2)	1.000
**DF4**	1 (0.2)	1 (0.2)	1 (0.3)	0 (0.0)	0 (0.0)	1 (0.6)	2 (0.2)	2 (0.2)	1.000
**DF5**	1 (0.2)	0 (0.0)	2 (0.6)	2 (0.6)	0 (0.0)	0 (0.0)	3 (0.3)	2 (0.2)	1.000
**ECU**	3 (0.7)	4 (1.0)	5 (1.4)	4 (1.1)	1 (0.6)	0 (0.0)	9 (1.0)	8 (0.9)	1.000
**TPD**									
**DF1**	0 (0.0)	1 (0.2)	0 (0.0)	0 (0.0)	1 (0.5)	0 (0.0)	1 (0.1)	1 (0.1)	1.000
**DF2**	0 (0.0)	0 (0.0)	0 (0.0)	1 (0.3)	0 (0.0)	0 (0.0)	0 (0.0)	1 (0.1)	-
**DF3**	1 (0.2)	0 (0.0)	0 (0.0)	0 (0.0)	0 (0.0)	0 (0.0)	1 (0.1)	0 (0.0)	-
**DF4**	0 (0.0)	0 (0.0)	0 (0.0)	0 (0.0)	0 (0.0)	1 (0.6)	0 (0.0)	1 (0.1)	-
**DF5**	0 (0.0)	0 (0.0)	0 (0.0)	0 (0.0)	0 (0.0)	0 (0.0)	0 (0.0)	0 (0.0)	-
**ECU**	0 (0.0)	0 (0.0)	0 (0.0)	0 (0.0)	0 (0.0)	0 (0.0)	0 (0.0)	0 (0.0)	-
**TEF**									
**DF1**	7 (1.7)	12 (3.0)	4 (1.1)	3 (0.8)	3 (1.6)	3 (1.6)	14 (1.5)	18 (1.9)	0.481
**DF2**	2 (0.5)	4 (1.0)	6 (1.7)	4 (1.1)	3 (1.6)	2 (1.1)	11 (1.2)	10 (1.1)	1.000
**DF3**	3 (0.7)	2 (0.5)	6 (1.7)	4 (1.1)	2 (1.1)	5 (2.7)	11 (1.2)	11 (1.2)	1.000
**DF4**	2 (0.5)	2 (0.5)	3 (0.8)	2 (0.6)	4 (2.2)	6 (3.2)	9 (1.0)	10 (1.1)	1.000
**DF5**	5 (1.2)	2 (0.5)	8 (2.2)	7 (1.9)	4 (2.2)	6 (3.2)	17 (1.8)	15 (1.6)	0.845
**ECU**	14 (3.5)	16 (4.)	9 (2.6)	9 (2.6)	3 (1.7)	6 (3.3)	26 (2.8)	31 (3.3)	0.442

HS, healthy subject; TSH, tenosynovial hypertrophy; TPD, power Doppler within tendon sheath; TEF, tenosynovial effusion; L, left; R, right. *Fisher's exact test

Trickey J, *et al. Ann Rheum Dis* 2022; 781:232–236. doi: 10.1136/annrheumdis-2021-219931

Supplementary table 6: Difference in proportion of TEF grade ≥1 compared to TSH grade ≥1 and TPD grade ≥1 in HS

	DF TSH grade ≥ 1 n (%)	DF TEF grade ≥ 1 n (%)	p value*
**Total number of tendons at each level**	1873	1873	
**DF1 TSH vs DF1 TEF**	2 (0.1)	32 (1.7)	0.000
**DF2 TSH vs DF2 TEF**	3 (0.2)	21 (1.1)	0.000
**DF3 TSH vs DF3 TEF**	5 90.3)	22 (1.2)	0.000
**DF4 TSH vs DF4 TEF**	4 (0.2)	19 (1.0)	0.001
**DF5 TSH vs DF5 TEF**	5 (0.3)	32 (1.7)	0.000
**ECU TSH vs ECU TEF**	17 (0.9)	57 (3.0)	0.000
**DF1 PD vs DF1 EF**	2 (0.1)	32 (1.7)	0.000
**DF2 PD vs DF2 EF**	1 (0.1)	21 (1.1)	0.000
**DF3 PD vs DF3 EF**	1 (0.1)	22 (1.2)	0.000
**DF4 PD vs DF4 EF**	1 (0.1)	19 (1.0)	0.000
**DF5 PD vs DF5 EF**	0 (0)	32 (1.7)	n/a
**ECU PD vs ECU EF**	0 (0)	57 (3.0)	n/a

TSH, tenosynovial hypertrophy; TPD, power Doppler within tendon sheath; TEF, tenosynovial effusion; DF, digit flexor tendon; ECU, extensor carpi ulnaris tendon. *McNemar's test

Supplementary table 7: Comparison of ECU tenosynovial hypertrophy with DF tendons 1-5 in healthy subjects

	ECU TSH grade ≥ 1 n (%)	DF TSH grade ≥ 1 n (%)	p value*
**Total number of tendons**	1867	1867	
**ECU TSH vs DF1 TSH**	17 (0.9)	2 (0.1)	0.001
**ECU TSH vs DF2 TSH**	17 (0.9)	3 (0.2)	0.003
**ECU TSH vs DF3 TSH**	17(0.9)	5 (0.3)	0.017
**ECU TSH vs DF4 TSH**	17 (0.9)	4 (0.2)	0.007
**ECU TSH vs DF5 TSH**	17 (0.9)	5 (0.3)	0.017

TSH, tenosynovial hypertrophy; TPD, power Doppler within tendon sheath; TEF, tenosynovial effusion; DF, digit flexor tendon; ECU, extensor carpi ulnaris tendon. *McNemar's test

Trickey J, *et al. Ann Rheum Dis* 2022; 781:232–236. doi: 10.1136/annrheumdis-2021-219931

Supplementary table 8: HS with grade ≥ 1 TSH, TPD and TEF in manual workers vs non manual workers

	Manual worker Tendon number (%)	Non manual worker Tendon number (%)	p value*
**Total number of tendons at each level**	136	1735	
**TSH**			
**DF 1 TSH G ≥ 1**	1 (0.7)	1 (0.1)	0.140
**DF 2 TSH G ≥ 1**	0 (0.0)	3 (0.2)	1.000
**DF 3 TSH G ≥ 1**	0 (0.0)	5 (0.3)	1.000
**DF 4 TSH G ≥ 1**	0 (0.0)	4 (0.2)	1.000
**DF 5 TSH G ≥ 1**	0 (0.0)	5 (0.3)	1.000
**ECU TSH G ≥ 1**	1 (1.5)	16 (0.9)	1.000
**TPD**			
**DF 1 TPD G ≥ 1**	1 (0.7)	1 (0.1)	0.140
**DF 2 TPD G ≥ 1**	0 (0.0)	1 (0.1)	1.000
**DF 3 TPD G ≥ 1**	0 (0.0)	1 (0.1)	1.000
**DF 4 TPD G ≥ 1**	0 (0.0)	1 (0.1)	1.000
**DF 5 TPD G ≥ 1**	0 (0.0)	0 (0.0)	n/a
**ECU TPD G ≥ 1**	0 (0.0)	0 (0.0)	n/a
**TEF**			
**DF 1 TEF G ≥ 1**	5 (3.7)	27 (1.6)	0.175
**DF 2 TEF G ≥ 1**	2 (1.5)	19 (1.1)	0.768
**DF 3 TEF G ≥ 1**	0 (0.0)	22 (1.3)	0.588
**DF 4 TEF G ≥ 1**	0 (0.0)	19 (1.1)	0.583
**DF 5 TEF G ≥ 1**	3 (2.2)	29 (1.7)	0.658
**ECU TEF G ≥ 1**	4 (2.9)	52 (3.0)	1.000

HS, healthy subject; G, grade; THS, tenosynovial hypertrophy; TPD, power Doppler within the tendon sheath; TEF, tenosynovial effusion; DF, digit flexor tendon; ECU, extensor carpi ulnaris tendon. * Fisher’s exact test

Supplementary table 9: Ultrasound tendon findings in healthy subjects with high impact vs low impact hobbies

	High impact upper limb hobbies Tendon Number (%)	Low impact upper limb hobbies Tendon Number (%)	p value*
**Total number of tendons at each level**	376	1502	
**TSH**			
**DF 1 TSH G ≥ 1**	1 (0.3)	1 (0.1)	0.361
**DF 2 TSH G ≥ 1**	0 (0.0)	3 (0.2)	1.000
**DF 3 TSH G ≥ 1**	0 (0.0)	5 (0.3)	0.590
**DF 4 TSH G ≥ 1**	0 (0.0)	4 (0.3)	0.590
**DF 5 TSH G ≥ 1**	0 (0.0)	5 (0.3)	0.590
**ECU TSH G ≥ 1**	0 (0.0)	17 (1.1)	0.033
**TPD**			
**DF 1 TPD G ≥ 1**	1 (0.3)	1 (0.1)	0.361
**DF 2 TPD G ≥ 1**	0 (0.0)	1 (0.1)	1.000
**DF 3 TPD G ≥ 1**	0 (0.0)	1 (0.1)	1.000
**DF 4 TPD G ≥ 1**	0 (0.0)	1 (0.1)	1.000
**DF 5 TPD G ≥ 1**	0 (0.0)	0 (0.0)	n/a
**ECU TPD G ≥ 1**	0 (0.0)	0 (0.0)	n/a
**TEF**			
**DF 1 TEF G ≥ 1**	3 (0.8)	29 (1.9)	0.199
**DF 2 TEF G ≥ 1**	1 (0.3)	20 (1.3)	0.145
**DF 3 TEF G ≥ 1**	1 (0.3)	21 (1.4)	0.123
**DF 4 TEF G ≥ 1**	1 (0.3)	18 (1.2)	0.189
**DF 5 TEF G ≥ 1**	3 (1.8)	29 (1.9)	0.199
**ECU TEF G ≥ 1**	18 (4.8)	39 (2.6)	0.049

HS, healthy subject; G, grade; THS, tenosynovial hypertrophy; TPD, power Doppler within the tendon sheath; TEF, tenosynovial effusion; DF, digit flexor tendon; ECU, extensor carpi ulnaris tendon. * Fisher's exact test

Trickey J, *et al. Ann Rheum Dis* 2022; 781:232–236. doi: 10.1136/annrheumdis-2021-219931

Supplementary table 10: Ultrasound tendon findings in age and sex matched healthy subjects and patients with RA

	HS tendons grade ≥1 Number (%)	RA tendons grade ≥1 Number (%)	p value* HS vs RA (age and sex matched)
**Total number of tendons at each level**	288	288	
**DF 1 TSH grade ≥1 n (%)**	0 (0)	15 (5.2)	< 0.001
**DF 2 TSH grade ≥1 n (%)**	0 (0)	50 (17.4)	< 0.001
**DF 3 TSH grade ≥1 n (%)**	1 (0.3)	50 (17.4)	< 0.001
**DF 4 TSH grade ≥1 n (%)**	1 (0.3)	28 (9.7)	< 0.001
**DF 5 TSH grade ≥1 n (%)**	5 (1,7)	36 (12.5)	< 0.001
**ECU TSH grade ≥1 n (%)**	6 (2.1)	60 (20.1)	< 0.001
**DF 1 TPD grade ≥1 n (%)**	0 (0)	10 (3.5)	0.002
**DF 2 TPD grade ≥1 n (%)**	0 (0)	36 (12.5)	< 0.001
**DF 3 TPD grade ≥1 n (%)**	0 (0)	40 (13.9)	< 0.001
**DF 4 TPD grade ≥1 n (%)**	0(0)	20 (6.9)	< 0.001
**DF 5 TPD grade ≥1 n (%)**	0 (0)	23 (8.0)	< 0.001
**ECU TPD grade ≥1 n (%)**	0 (0)	58 (20.3)	< 0.001

HS, healthy subject; THS, tenosynovial hypertrophy; TPD, power Doppler within the tendon sheath; TEF, tenosynovial effusion; DF, digit flexor tendon; ECU, extensor carpi ulnaris tendon. * Fisher's exact test.

Trickey J, *et al. Ann Rheum Dis* 2022; 781:232–236. doi: 10.1136/annrheumdis-2021-219931
