# Supplementary figures and images for: Very low prevalence of ultrasound-detected tenosynovial abnormalities in healthy subjects throughout the age range: OMERACT ultrasound minimal disease study
Source: Ann Rheum Dis. 2021 Aug 18;81(2):232–6. doi: 10.1136/annrheumdis-2021-219931 (PMC8762026; doi:10.1136/annrheumdis-2021-219931)

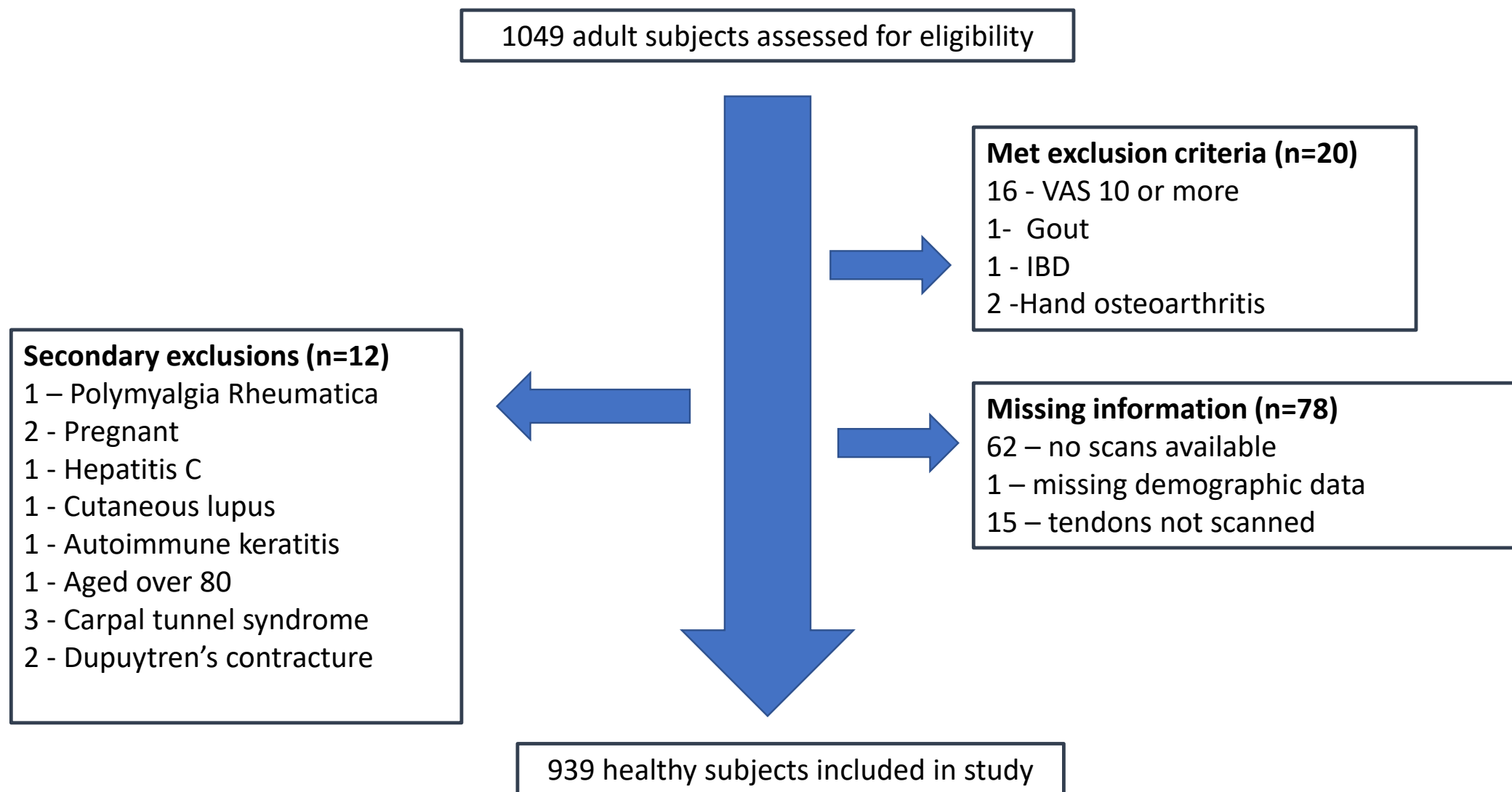

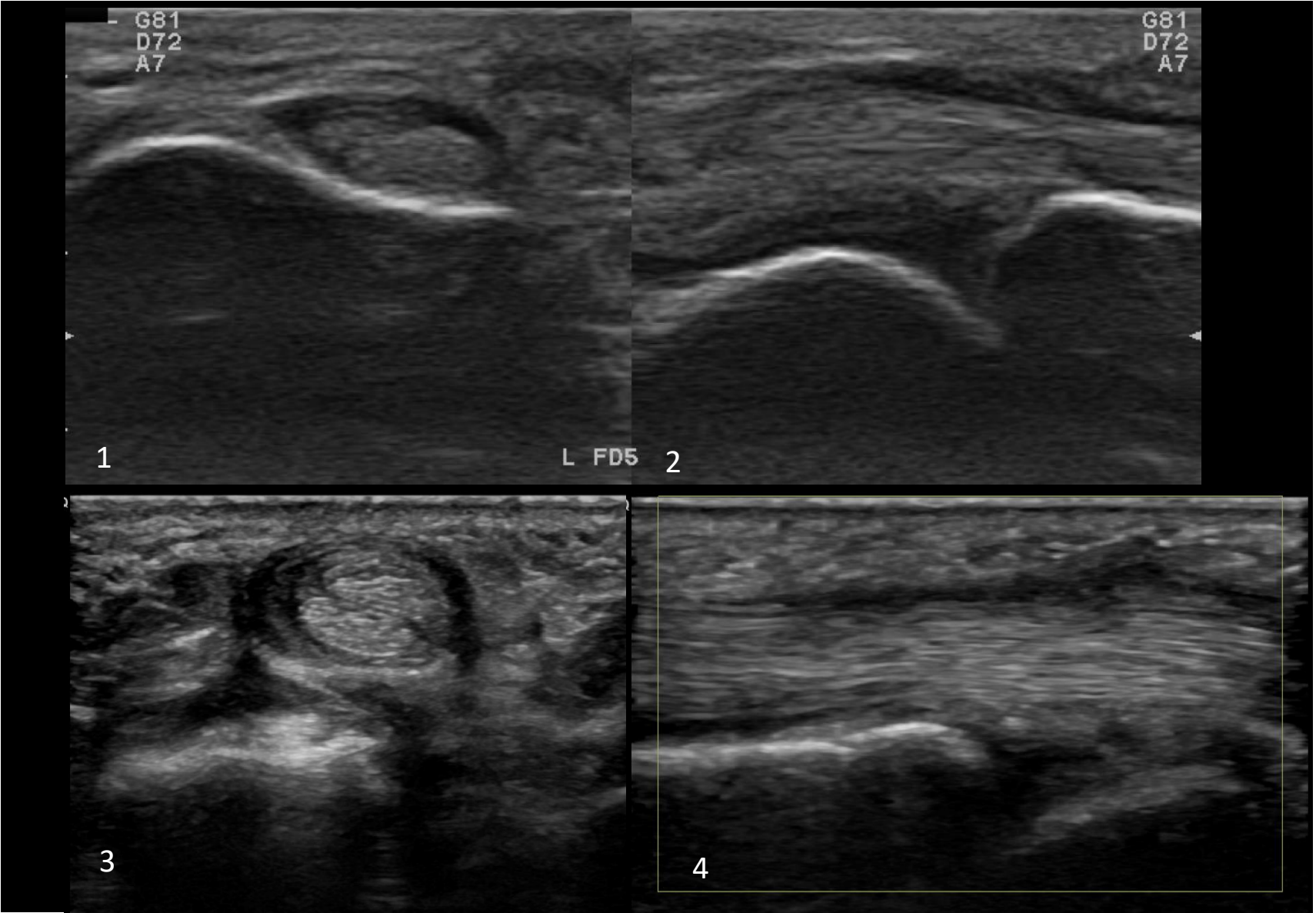

Supplement: Supplementary data [file annrheumdis-2021-219931supp001.pdf]
