# Supplementary material for: Very low prevalence of ultrasound-detected tenosynovial abnormalities in healthy subjects throughout the age range: OMERACT ultrasound minimal disease study
Source: Ann Rheum Dis. 2021 Aug 18;81(2):232–6. doi: 10.1136/annrheumdis-2021-219931 (PMC8762026; doi:10.1136/annrheumdis-2021-219931)
Supplement: Supplementary data [file annrheumdis-2021-219931supp003.pdf]

Supplementary Figure 1: Flow chart diagram of inclusion and exclusion of healthy subjects

Supplementary Figure 2: Example images of tenosynovial hypertrophy, tenosynovial effusion within the tendon sheath in healthy subjects and patients with Rheumatoid Arthritis

1. Transverse section of left digit flexor 3 in a healthy subject showing tenosynovial hypertrophy and effusion
2. Longitudinal section of left digit flexor 3 in a healthy subject showing tenosynovial hypertrophy and effusion
3. Transverse section of right digit flexor 2 in a patient with Rheumatoid arthritis showing tenosynovial hypertrophy and effusion
4. Longitudinal section of right digit flexor 2 in a patient with Rheumatoid arthritis showing tenosynovial hypertrophy and effusion
